# Office Visitor

**Published:** 1906-06

**Authors:** 


### OFFICE VISITOR.

She was an old friend of the family. Kind and faithful in her friendship, but very willful, having a high opinion of her worth and superior judgment and inclined at times to be a bit nettled when crossed in action or argument. She was a nurse of ability, trained in the school of experience, before there were regular training schools. Prejudiced against some simple things and eccentric as she aged, she had had the bloom of youth once in her faded cheeks and was said to have been good looking and of fine carriage. She had had a soldier boy sweetheart who was wounded in battle and she nursed him while he languished and died on the hospital cot. She then and there devoted her life to nursing the sick and the injured. Though she assisted and saw many operations, she never could summon the nerve to have her teeth operated on or extracted. It was therefore after much persuasion by her relatives that she applied to us for a set of teeth and only consented to the taking of the impression—"if you don't pull any of my teeth, just grind the roots down and put the set in just so." The work was done just so, and but for mutual regard an open rupture would have occurred between the painstaking but hampered dentist and his apparently intentionally fault-finding and obtuse patient. "They won't stick up and when I bite on 'em my gums bleed, and I can't bite anything hard—they rock in the mouth and come down." Explanation about the old roots being the impediment in the way of their "going up" brought the reply, "Well, you are not going to pull them out, I tell you now, I'll die if you try it and I'm not going to let you try." After a siege of visits and "working on them," she at last "got used to them." During two years' wear the plate forced loose and out of sockets several roots and in consequence of a lack of support several teeth broke from the plate and at last the latter cracked and split in two. A visit to us resulted. "I have taken a great deal of pride in you and have always told my friends that you were a fine dentist and that your work always lasted, but you have done me a miserable piece of work and your old plate is all to pieces." We explained that the loss of the roots caused the loss of the teeth and the splitting by lack of support and advised reinforcement with a rim of pink rubber in the replacing of the teeth. "No, I don't want any of that stuff showing in my mouth—fix them as you did at first but make them to stand—give me good strong work." Argument ensued and the rim was put on, but by some fault of the assistant there was a disarrangement of the teeth (it always occurs in such cases, when it is most desirable that everything shall go right to prove your almost sworn statements). This she quickly discovered and though it made little difference she blessed us out and declared she "would be back in a short time to have them done all over." She nursed her wrath and came back at appointment and assured us that had we not been such old friends she would never darken the door of the office again. Purposely, we assured her that we could not if we tried a hundred times, do better than we did unless the remaining teeth were out. Immediately she altered her tone and said, "Now you know I can't stand that and really they are doing better than they did at first and do you know my folks say the little change in the setting of the teeth becomes me—so I guess I'll let them stay as they are. I thank you very much—forgive me if I've been nervous and a little cross to you—you always would have your way with me but I guess it has been best for me. How's your family? Give 'em my love. Good bye."

